# Quantitative ^13^C‐isotope labelling‐based analysis to elucidate the influence of environmental parameters on the production of fermentative aromas during wine fermentation

**DOI:** 10.1111/1751-7915.12749

**Published:** 2017-07-11

**Authors:** Stéphanie Rollero, Jean‐Roch Mouret, Audrey Bloem, Isabelle Sanchez, Anne Ortiz‐Julien, Jean‐Marie Sablayrolles, Sylvie Dequin, Carole Camarasa

**Affiliations:** ^1^ UMR SPO: INRA, Universite Montpellier Montpellier SupAgro 34060 Montpellier France; ^2^ Lallemand SAS 31700 Blagnac France; ^3^ Present address: Institute for Wine Biotechnology Department of Viticulture and Oenology Stellenbosch University Private Bag X1 Matieland 7602 South Africa

## Abstract

Nitrogen and lipids are key nutrients of grape must that influence the production of fermentative aromas by wine yeast, and we have previously shown that a strong interaction exists between these two nutrients. However, more than 90% of the acids and higher alcohols (and their acetate ester derivatives) were derived from intermediates produced by the carbon central metabolism (CCM). The objective of this study was to determine how variations in nitrogen and lipid resources can modulate the contribution of nitrogen and carbon metabolisms for the production of fermentative aromas. A quantitative analysis of metabolism using ^13^C‐labelled leucine and valine showed that nitrogen availability affected the part of the catabolism of N‐containing compounds, the formation of α‐ketoacids from CCM and the redistribution of fluxes around these precursors, explaining the optimum production of higher alcohols occurring at an intermediate nitrogen content. Moreover, nitrogen content modulated the total production of acids and higher alcohols differently, through variations in the redox state of cells. We also demonstrated that the phytosterol content, modifying the intracellular availability of acetyl‐CoA, can influence the flux distribution, especially the formation of higher alcohols and the conversion of α‐ketoisovalerate to α‐ketoisocaproate.

## Introduction

Controlling the quality of wines is a major issue in the winemaking sector: in an increasingly competitive market, a key objective is to best meet consumer expectations in terms of the organoleptic profile of the product (Lee and Noble, 2003; Swiegers *et al*., 2005). Therefore, one of the current major challenges is to optimize the quality of wine, including its properties on a sensory level. The aroma of a wine is defined by a complex mixture of molecules of different origins: varietal aromas and their precursors coming from grapes, fermentative aromas synthesized by yeast and postfermentative aromas released during the ageing of wines. In this study, we are particularly interested in fermentative aroma compounds, which include molecules belonging to different chemical families (higher alcohols, acetate esters, medium‐chain fatty acids and ethyl esters). Their syntheses involve a highly interconnected metabolic network (Swiegers *et al*., 2005). These volatile compounds can be produced from very different metabolic pathways, e.g. central carbon metabolism (CCM), lipid metabolism or amino acid catabolism (nitrogen metabolism); furthermore, some of these fermentative aromas are derived from common precursors, while others share enzymes involved in their metabolic routes (e.g. higher alcohols). In accordance with this complexity, it was shown that the production of fermentative aromas depends on many environmental factors (assimilable nitrogen, availability of lipids, temperature, etc.; Hernandez‐Orte *et al*., 2006; Varela *et al*., 2012; Mouret *et al*., 2014; Rollero *et al*., 2015).

Several studies have assessed the influence of assimilable nitrogen content on the production of fermentative aromas (reviewed in Bell and Henschke, 2005). Generally, a direct relationship between initial nitrogen content and higher alcohol concentrations was observed when the nitrogen content remained low, whereas at moderate to high nitrogen quantities, there was an inverse relationship (Jiménez‐Martí *et al*., 2007; Vilanova *et al*., 2007, 2012; Carrau *et al*., 2008; Mouret *et al*., 2014; Rollero *et al*., 2015). Acetates and ethyl esters showed a more simple relationship with the nitrogen concentration: an increase in initial nitrogen content was associated with an increase in ester production (Hernandez‐Orte *et al*., 2006; Garde‐Cerdán and Ancín‐Azpilicueta, 2008; Ugliano *et al*., 2010; Torrea *et al*., 2011; Mouret *et al*., 2014; Rollero *et al*., 2015). Conversely, the influence of lipids and sterols on the synthesis of these molecules has been little studied. Sterols and fatty acids not only are essential components for maintaining the integrity of the yeast membrane but also are involved as precursors in the synthesis of certain aroma compounds (Swiegers *et al*., 2005; Styger *et al*., 2011; Varela *et al*., 2012). When exogenous unsaturated fatty acids are available in abundance, cells incorporate them into their membrane, causing a significant reduction in the *de novo* synthesis of fatty acids. Accordingly, the production of aroma compounds such as ethyl esters and isoamyl acetate is reduced (Yunoki *et al*., 2005). These observations were confirmed by Saerens *et al*. (2008), who showed that the addition of unsaturated fatty acids into fermentation medium causes a decrease in the production of ethyl esters and acetate esters. This is consistent with the ability of ergosterol to inhibit the expression of the *ATF1* gene, which is the main gene coding for the alcohol acetyltransferase responsible for the production of the majority of acetate esters (Malcorps and Dufour, 1992; Fujii *et al*., 1997; Fujiwara *et al*., 1998). Moreover, it has been reported that an increase in availability of phytosterols, which are unsaturated fatty acids found in grape must, seemed to favour the formation of higher alcohols but caused a decrease in the production of esters (Rollero *et al*., 2015).

In this context, to define strategies to modulate the organoleptic characteristics of wines, it is necessary to better understand the effect of environmental parameters on the metabolic origins of fermentative aromas. These aspects are difficult to capture only by monitoring the metabolites produced, and isotopic filiation approaches have been identified as one of the tools of choice to deepen metabolic knowledge (Blank *et al*., 2005; Frick and Wittmann, 2005; Christen and Sauer, 2011). An overview of the management of nitrogen by yeast during conventional wine fermentation has recently been provided from an experimental design based on a combination of ^13^C and ^15^N tracer experiments. This approach revealed that the incorporation of exogenous amino acids in biomass was limited in favour of *de novo* synthesis under the study conditions. In addition, the catabolism of amino acids plays a minor role in the formation of volatile compounds, with the α‐ketoacids precursors required for these neo‐syntheses mainly originating from the carbon metabolism (Crépin *et al*., 2017).

The objective of this study was to determine how variations in nitrogen and lipid resources can modulate the contributions of both nitrogen and carbon metabolisms to the production of fermentative aromas using filiation experiments with ^13^C‐labelled nitrogen sources. Understanding this relationship provided deeper knowledge on how environmental factors (initial nitrogen and phytosterol content) can influence the contribution of nitrogen and carbon metabolisms to the production of fermentative aromas.

## Results

To assess the distribution of nitrogen sources in proteinogenic amino acids and volatile compounds, we used a quantitative approach based on stable isotope tracer experiments with amino acids labelled on their carbon skeleton. This ^13^C labelling provides information on the amino acid fractions directly used for biomass formation and those leading to the formation of fermentative aromas, which would not be possible using a ^15^N‐labelling strategy. Leucine and valine, which are precursors of the most abundant higher alcohols in wines – isoamyl alcohol and isobutanol, respectively – and which have interconnected metabolic pathways, were used in this study.

To study the effect of environmental conditions on the flux partitioning in the whole metabolic network, fermentations were carried out with a different initial nitrogen (70, 250 or 425 mg l^−1^) and phytosterol (2 or 8 mg l^−1^) content using a commercial wine yeast strain, Lalvin EC1118^®^. The different nitrogen concentrations were made up by a mixture of ammonium and amino acids, that kept the same proportion. For each fermentation, only a single nitrogen compound was supplied as a labelled molecule, either valine or leucine. These amino acids, each accounting for 1.3% of total yeast assimilable nitrogen, are fully consumed by yeasts during fermentation, regardless of nitrogen availability (between 70 to 425 mg N l^−1^). Because nitrogen sources are sequentially assimilated during the growth phase of wine fermentation (Crepin *et al*., 2012), we investigated the flux partitioning at different stages of the culture corresponding to a partial (N_1/2_ and N_3/4_) or complete consumption of the nitrogen content (N_T_) and at the end of fermentation (EF). We measured the ^13^C isotopic enrichment of proteinogenic amino acids and of volatile compounds (higher alcohols, their acetate esters and acids) that may be synthetized from leucine and valine through the Ehrlich pathway (Hazelwood *et al*., 2008).

### Origin of the carbon backbone of proteinogenic valine and leucine

**Figure 1:**
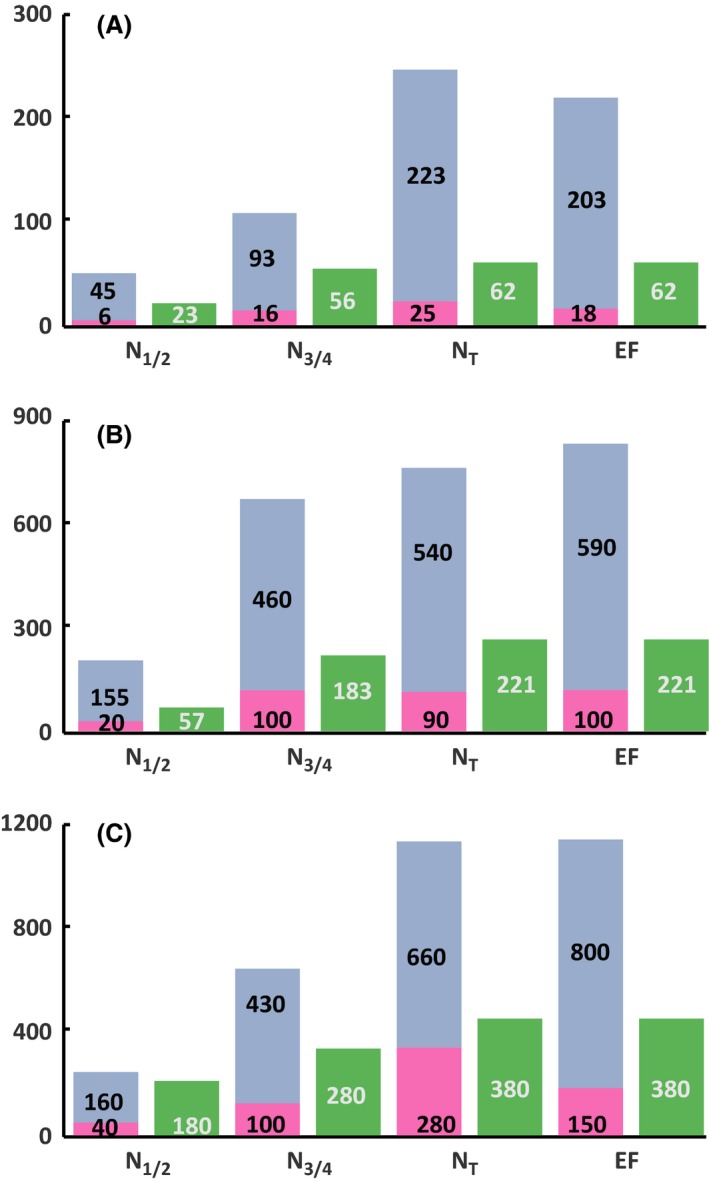
Comparison of the concentration (μM) of consumed valine (green) and proteinogenic valine from *de novo* synthesis (blue) and from direct incorporation of consumed valine (pink) at the four stages of fermentation (N_1/2_, N_3/4_, N_T_ and EF) in SM70 (A), SM250 (B) and SM425 (C) at 2 mg/l phytosterols.

**Figure 2:**
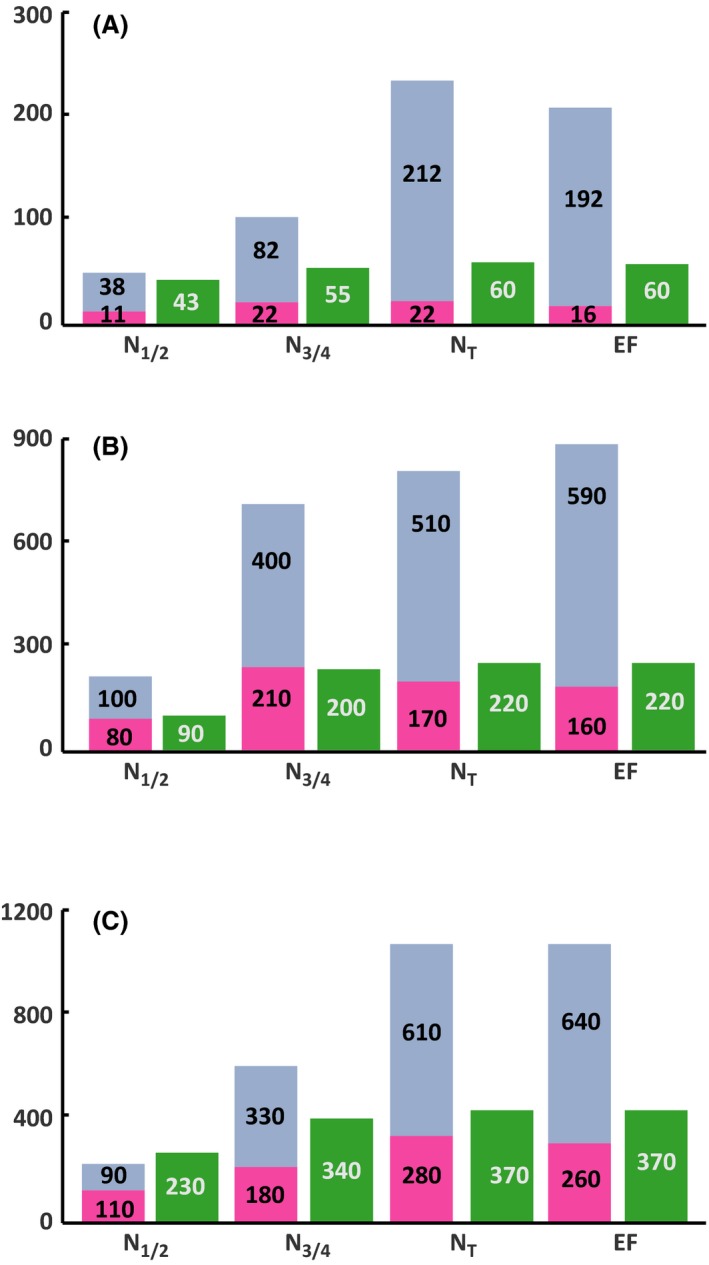
Comparison of the amount (μM) of consumed leucine (green) and proteinogenic leucine from *de novo* synthesis (blue) and from direct incorporation of consumed leucine (pink) at the four stages of fermentation (N_1/2_, N_3/4_, N_T_ and EF) in SM70 (A), SM250 (B) and SM425 (C) at 2 mg l^−1^ phytosterols.

**Figure 3:**
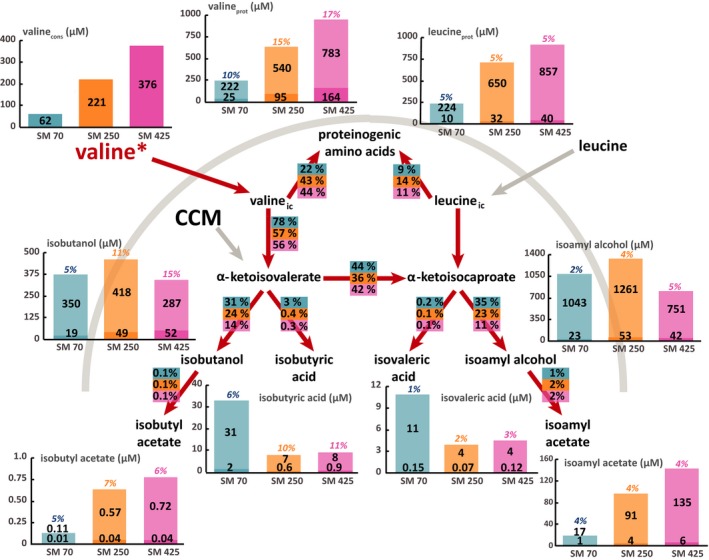
Flux partitioning around the valine metabolism at 2 mg l^−1^ phytosterols and three different levels of assimilable nitrogen. The distribution of fluxes around these metabolic routes was investigated using isotopic filiation experiments with ^13^C‐labelled valine. Portion of consumed valine recovered in proteinogenic leucine and valine: the labelled fraction (light colour) corresponds to the consumed valine directly incorporated into proteins, while the unlabelled fraction (dark colour) represents the proteinogenic amino acids *de novo* synthetized from CCM precursors. Portion of the valine consumed converted into volatile compounds: the labelled fraction (light colour) corresponds to the fraction of volatile compounds synthetized using carbon from consumed valine, while the unlabelled fraction (dark colour) represents the portion of volatile molecules synthetized from α‐ketoacids through the CCM. Flux partitioning involved in the use of valine during fermentation: the fraction of consumed valine catabolized through a certain pathway was assessed from the molar ratios between the amount of a proteinogenic amino acid or volatile compound labelled and the total amount of consumed amino acid. Isotopic enrichments (defined as the molar ratio between the quantity of labelled compound and the total quantity) are shown above each bar plot. The raw data and details of the calculations are provided in the Supplemental Material. Blue: SM70; orange: SM250; pink: SM425.

**Figure 4:**
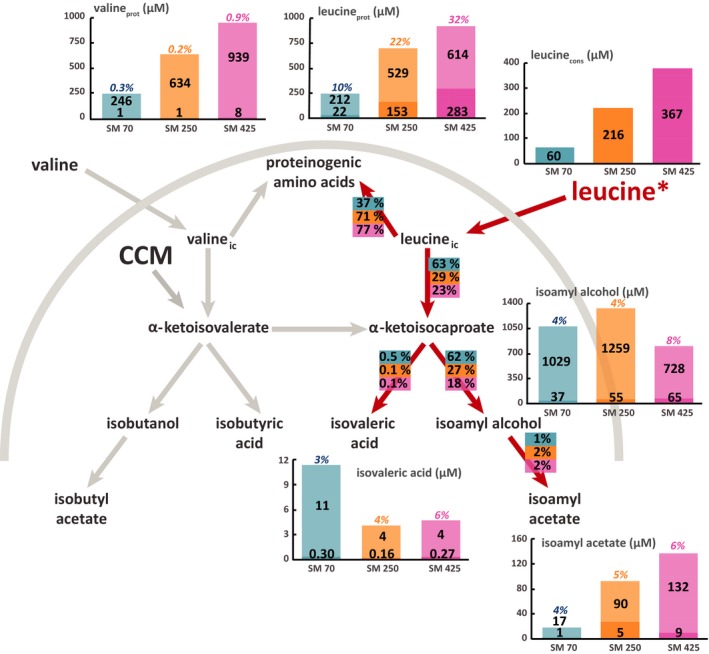
Flux partitioning around the leucine metabolism at 2 mg l^−1^ phytosterols and three different levels of assimilable nitrogen. The distribution of fluxes around these metabolic routes was investigated using isotopic filiation experiments with ^13^C‐labelled leucine. Portion of consumed leucine recovered in proteinogenic leucine: the labelled fraction (light colour) corresponds to the consumed leucine directly incorporated into proteins, while the unlabelled fraction (dark colour) represents the proteinogenic amino acids *de novo* synthetized from CCM precursors. Portion of consumed leucine converted into volatile compounds: the labelled fraction (light colour) corresponds to the fraction of volatile compounds synthetized using carbon from consumed leucine, while the unlabelled fraction (dark colour) represents the portion of volatile molecules synthetized from α‐ketoacids through the CCM. Flux partitioning involved in the use of leucine during fermentation: the fraction of consumed leucine catabolized through a certain pathway was assessed from the molar ratios between the amount of a proteinogenic amino acid or volatile compound labelled and the total amount of consumed amino acid. Isotopic enrichments (defined as the molar ratio between the quantity of labelled compound and the total quantity) are shown above each bar plot. The raw data and details of the calculations are provided in the Supplemental Material. Blue: SM70; orange: SM250; pink: SM425.

**Figure 5:**
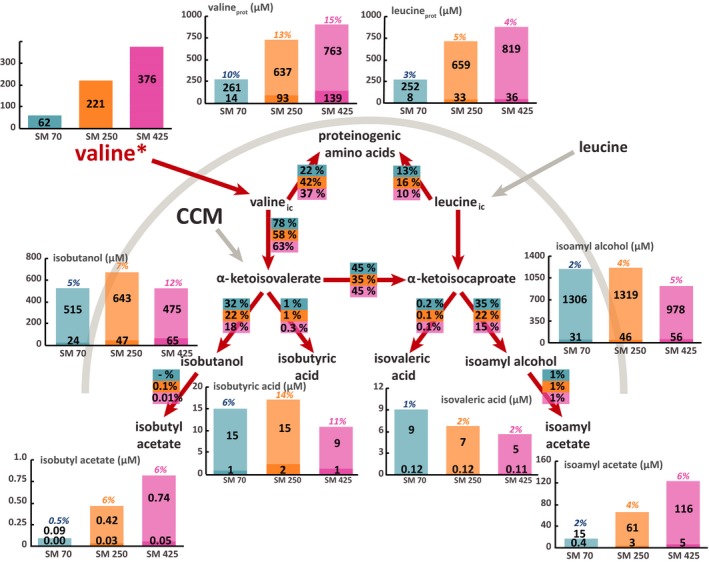
Flux partitioning around the valine metabolism at 8 mg l^−1^ phytosterols and three different levels of assimilable nitrogen. The distribution of fluxes around these metabolic routes was investigated using isotopic filiation experiments with ^13^C‐labelled valine. Portion of consumed valine recovered in proteinogenic leucine and valine: the labelled fraction (light colour) corresponds to the consumed valine directly incorporated into proteins, while the unlabelled fraction (dark colour) represents the proteinogenic amino acids *de novo* synthetized from CCM precursors. Portion of consumed valine converted into volatile compounds: the labelled fraction (light colour) corresponds to the fraction of volatile compounds synthetized using carbon from consumed valine, while the unlabelled fraction (dark colour) represents the portion of volatile molecules synthetized from α‐ketoacids through the CCM. Flux partitioning involved in the use of valine during fermentation: the fraction of consumed valine catabolized through a certain pathway was assessed from the molar ratios between the amount of a proteinogenic amino acid or volatile compound labelled and the total amount of consumed amino acid. Isotopic enrichments (defined as the molar ratio between the quantity of labelled compound and the total quantity) are shown above each bar plot. The raw data and details of the calculations are provided in the Supplemental Material. Blue: SM70; orange: SM250; pink: SM425.

**Figure 6:**
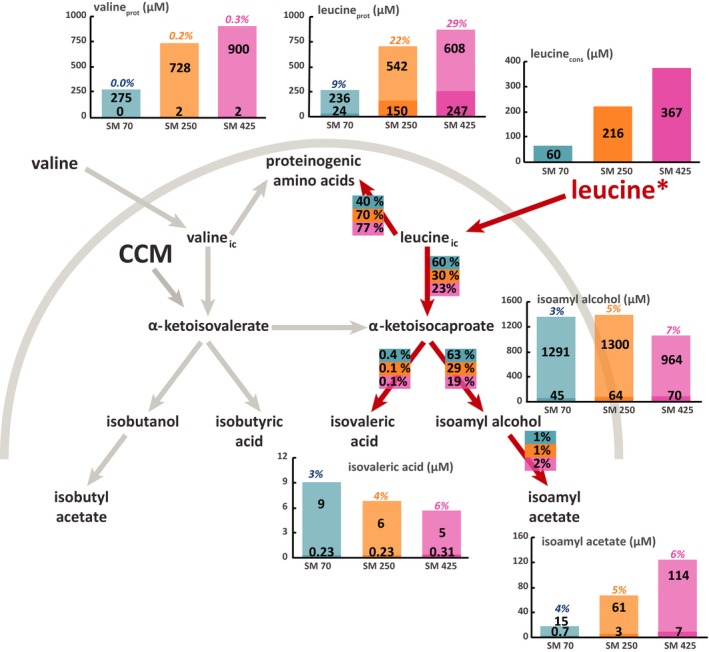
Flux partitioning around the leucine metabolism at 8 mg l^−1^ phytosterols and three different levels of assimilable nitrogen. The distribution of fluxes around these metabolic routes was investigated using isotopic filiation experiments with ^13^C‐labelled leucine. Portion of consumed leucine recovered in proteinogenic leucine: the labelled fraction (light colour) corresponds to the consumed leucine directly incorporated into proteins, while the unlabelled fraction (dark colour) represents the proteinogenic amino acids *de novo* synthetized from CCM precursors. Portion of consumed leucine converted into volatile compounds: the labelled fraction (light colour) corresponds to the fraction of volatile compounds synthetized using carbon from consumed leucine, while the unlabelled fraction (dark colour) represents the portion of volatile molecules synthetized from α‐ketoacids through the CCM. Flux partitioning involved in the use of leucine during fermentation: the fraction of consumed leucine catabolized through a certain pathway was assessed from the molar ratios between the amount of a proteinogenic amino acid or volatile compound labelled and the total amount of consumed amino acid. Isotopic enrichments (defined as the molar ratio between the quantity of labelled compound and the total quantity) are shown above each bar plot. The raw data and details of the calculations are provided in the Supplemental Material. Blue: SM70; orange: SM250; pink: SM425.

In the beginning, we quantified the fraction of valine and leucine present in the biomass that originates directly from exogenous amino acids. First, in the case of both amino acids, we observed a substantial imbalance early in the growth phase between the level of consumption of the exogenous amino acids and the content of these compounds in proteins that was unrelated to the nitrogen and phytosterol content (Figs 1 and 2). For example, the concentration of leucine in the medium was between 0.060 and 0.367 mmol l^−1^, while the content of this amino acid in the biomass varied between 0.230 and 0.903 mmol l^−1^ for SM70 and SM425 respectively. Overall, the proteinogenic amino acid content in cells was up to three to four times higher than the assimilated content (Figs 1 and 2). In accordance with these data, at the end of the growth phase (when the nitrogen was completely consumed), the isotopic enrichment of each amino acid that corresponded to the amount directly originating from the exogenous compound (Figs 3, 4, 5, 6, Tables SD1 and SD2) was lower than 35% at all nitrogen concentrations. All these observations indicate that the *de novo* synthesis of amino acids from the CCM plays an important role in fulfilling anabolic demand, even in a nitrogen‐rich environment. These observations generalize the observations made by Crépin *et al*. (2017) for only one content of assimilable nitrogen to a wider range of N availability. Furthermore, unexpectedly and under all environmental conditions, the direct incorporation of consumed amino acids in proteins was limited, despite the extent of anabolic requirements (at least two to three times higher than the concentration provided in the medium). This finding suggests that an important fraction of the consumed amino acids was catabolized by the yeast. In agreement with this hypothesis, ^13^C labelling was detected in leucine and in isobutanol and isoamyl alcohol in cultures with labelled valine (Figs 3 and 5, Tables SD1 and SD2).

Furthermore, regardless of the environmental conditions, we observed a greater decrease during the growth phase in the isotopic enrichment of leucine (a two‐ to threefold reduction), which was imported into the cells early on (Jiranek *et al*., 1995; Crepin *et al*., 2012), compared with that of valine (from 14% to 11%), which was consumed later (Tables SD1 and SD2). These observations revealed that the fraction of consumed amino acids directly recovered from the biomass was related to their sequential consumption.

### Impact of environmental parameters on the direct recovery of consumed leucine and valine in proteins

Overall, the partitioning between the fractions of exogenous amino acids directly incorporated in biomass and the part that is further catabolized was not influenced by phytosterol availability (Figs 3, 4, 5, 6, Tables SD1 and SD2). By contrast, we found that this distribution differed on the basis of the initial nitrogen content. For both amino acids (leucine and valine), we observed that the amount of labelled amino acid directly incorporated in proteins at the end of the growth phase was higher in SM250 and SM425 than in SM70 (Figs 3, 4, 5, 6, Tables SD1 and SD2). Nevertheless, the fraction directly recovered in the biomass also depended on the nature of the amino acid. In SM250 and SM425, the amount of labelled leucine in proteins was quite similar to the amount consumed (70–80%), while only 40% of the valine consumed was found in the biomass. In SM70, the exogenous leucine in the biomass accounted for approximately 40% of the consumed amount and around 20–30% for valine.

The non‐negligible and constant (approximately 4–5%) isotopic enrichment of leucine during fermentation in the presence of labelled valine (Figs 3 and 5) – by contrast, no valine enrichment was observed in the presence of labelled leucine (Figs 4 and 6) – is consistent with the existence of a leucine biosynthetic pathway from the carbon backbone of valine. Moreover, the fraction of valine catabolized through this metabolic route varied depending on the initial nitrogen content: in SM70 and SM425, 42–45% of consumed valine was converted into α‐ketoisocaproate, while only 35–36% was metabolized in SM250.

### Impact of environmental parameters on the metabolic origin of higher alcohols

Low isotopic enrichments of isobutanol (between 5% and 15%) and isoamyl alcohol (between 2% and 8%) were found in the presence of labelled valine and leucine irrespective of the initial nitrogen and phytosterol content (Figs 3, 4, 5, 6). These enrichments decreased throughout the fermentation process (Tables SD1 and SD2), indicating a strong contribution of the CCM to the formation of the ketoacid precursors of higher alcohols, which increased as the exogenous amino acids were depleted. The relationship between nitrogen content and higher alcohol concentration was not monotonic: there was a direct relationship at low nitrogen content (between 70 and 250 mg l^−1^), whereas an inverse relationship was found at moderate to high nitrogen concentrations (from 250 to 425 mg l^−1^). It was also interesting to note that the isotopic enrichment in isobutanol and isoamyl alcohol increased with the initial nitrogen content, even if the fraction of consumed amino acids recovered in these volatile compounds decreased. All of these results suggested that the contribution of ketoacids originating from CCM to the synthesis of higher alcohols varied according to the initial nitrogen content of the medium, with a higher contribution under conditions of nitrogen limitation. We also observed an enrichment of isoamyl alcohol in fermentations conducted with ^13^C‐valine (Figs 3 and 5). This finding confirmed that a part of the valine consumed was transformed into the α‐ketoisocaproate precursor of isoamyl alcohol through the CCM.

Overall, the phytosterol availability modulated the formation of higher alcohols (Figs 3, 4, 5, 6). For example, in SM425 at the lower phytosterol content, the concentration of isobutanol was 0.339 mM, while at 8 mg l^−1^ phytosterols, it was 0.540 mM. However, the extent of the response to changes in the phytosterol availability also depended on other factors, such as the nature of the higher alcohol and the nitrogen content. Thus, an increase of approximately 20% in the formation of isoamyl alcohol occurred regardless of the initial nitrogen content, while the increase in the amount of isobutanol synthesized with respect to the phytosterol availability varied from approximately 46–47% (SM70‐SM250) to 59% (SM425). The comparison between the labelling patterns of volatile compounds produced at low and high phytosterol content revealed the origin of the carbon backbone used to increase the formation of higher alcohols. We first observed that the amount of isobutanol produced from labelled valine tended to increase together with the concentration of phytosterols: in SM425, this amount was equal to 0.052 and 0.065 mM at 2 and 8 mg l^−1^ phytosterols respectively (Figs 3 and 5). This finding indicated that a larger fraction of valine was catabolized towards higher alcohols in the presence of high phytosterol content. However, the decrease in the isotopic enrichment of isobutanol with the increase in initial phytosterol content reflected a higher increase in the total formation of this compound than that of its labelled form. Consequently, the synthesis of α‐ketoisovalerate through the CCM appears to be the major contributor to the increase in the isobutanol production in response to changes in phytosterol availability. By contrast, no substantial modifications were observed in the isotopic enrichment of isoamyl alcohol from labelled leucine between fermentations with high or low initial phytosterol content. For this compound, increased phytosterol availability resulted in an increase in its flux of production without affecting the relative contributions of the CCM and of the catabolism of leucine into ketoacid precursors. These observations suggest that different regulatory effects triggered by the phytosterol availability control the biosynthetic pathways of these two higher alcohols.

### Impact of environmental parameters on the metabolic origin of acetate esters and acids

As observed in the case of the higher alcohols, low isotopic enrichments of acetate esters (7% maximum) were found in the presence of labelled valine and leucine irrespective of the initial content of nitrogen and phytosterols. Overall, the isotopic enrichment of isoamyl acetate was largely similar to that measured in its higher alcohol precursor (Figs 4 and 6). This finding is consistent with the fact that acetate esters can be produced from only higher alcohols without additional contributions from other pathways. A similar trend emerged for isobutyl acetate, but it was more difficult to observe because the concentrations were very low; therefore, the assays were less accurate. However, the formation of acetate esters and their corresponding higher alcohols was affected differently by the fermentative parameters (Figs 3, 4, 5, 6, Tables SD1 and SD2). The concentration of acetate esters increased together with the nitrogen content in the medium (e.g. at 2 mg l^−1^ phytosterols, isoamyl acetate was at a concentration of 18 μM in SM70 and 141 μM in SM425), as previously reported in the literature. Regarding the impact of the phytosterol content, a low lipid content was found to be more favourable for the production of acetate esters. This information suggests that the production of acetate esters is more dependent on the enzymatic activity of alcohol acetyltransferases (Atf1 and Atf2) than on the availability of higher alcohols. Indeed, a high lipid content downregulated the expression of the *ATF1* gene (Saerens *et al*., 2008).

Comparing the isovaleric and isobutyric acid concentrations in relation to the nitrogen and phytosterol availability clearly highlighted the ability of these environmental parameters to control the production of acids (Figs 3, 4, 5, 6). Overall, the production of acids decreased together with the increase in initial nitrogen content, except for the increase in isobutyric acid formation between SM70 and SM250. By contrast, the impact of increasing the initial phytosterol concentration (2 mg l^−1^) was dependent on nitrogen availability, with a decrease in acid production at low nitrogen content (SM70) and an increase in their formation in SM250 and SM425. The production of isobutyric acid exhibited greater variations than the production of isovaleric acid. Moreover, it is noteworthy that the response to changes in nutrient availability with regard to the production of acids differed from that of the synthesis of higher alcohols, as described above. Regarding the isotopic enrichments, the labelling incorporation found in acids presented the same pattern as was observed in the case of the higher alcohols: the isotopic enrichments remained low and tended to increase together with the nitrogen content (from 5% to 11% in SM70 and SM425, respectively), but we did not observe a significant impact of the lipid dose.

Together, these results are consistent with the fact that both acids and their corresponding higher alcohols were produced from the same ketoacids (Hazelwood *et al*., 2008), with a further distribution of these alcohol precursors towards acids depending on the nutrient composition of the medium.

Finally, we observed an enrichment of isovaleric acid in fermentations conducted with ^13^C‐valine (Figs 3 and 5), confirming the conversion of a part of the consumed valine into the α‐ketoisocaproate intermediate.

## Discussion

In yeast, α‐ketoacids play a key metabolic role at the interface between the CCM and the nitrogen metabolism. In fact, these intermediates may both originate from the CCM and be directly derived from the amino acid catabolism via transamination. Furthermore, they have been reported to be the main precursors in the anabolism of amino acids (Albers *et al*., 1996) and the starting point in the synthesis of branched acids, higher alcohols and their acetate derivatives through the Ehrlich pathway (Hazelwood *et al*., 2008). Under defined conditions (180 mg l^−1^ assimilable nitrogen and 2 mg l^−1^ ergosterol), a recent study has elucidated the fluxes distributed around the α‐ketoacids that are the metabolic origin of these compounds – relative to the contribution of CCM and amino acids catabolism – and their allocation (Crépin *et al*., 2017). In this work, we demonstrated how the partitioning around two α‐ketoacids nodes, α‐ketoisovalerate and α‐ketoisocaproate, varies depending on two environmental factors identified as modulators of biomass formation and fermentative aroma production: nitrogen and phytosterol availability.

First, our data underlined that the important contribution of catabolism to the fate of consumed amino acids – a feature that was unexpected until recently (Crépin *et al*., 2017) – was found regardless of the nitrogen and phytosterol content in the medium. However, we observed that only a small fraction of higher alcohols were synthetized using the carbon skeletons of amino acids. This could be explained by the fact that the quantity of amino acids consumed is often much lower than the amount of the corresponding higher alcohol produced. Consequently, most of these volatile compounds originated from the backbones of compounds produced through the CCM.

Concerning the influence of the nitrogen content, part of the catabolism of N‐containing compounds increased with the decrease in nitrogen availability. This finding is particularly relevant for leucine, one of the most abundant amino acids in biomass (Lange and Heijnen, 2001). In media containing moderate to high concentrations of nitrogen, direct incorporation into biomass accounted for approximately 70% of the consumed leucine. This behaviour, which is specific to this branched amino acid, may be explained by (i) its poor ability to support yeast growth as the sole nitrogen source (Godard *et al*., 2007), and (ii) its early importation into cells through the SPS‐controlled transporters Bap2p and Bap3p (Iraqui *et al*., 1999; Gaber *et al*., 2003), which occurs before the initiation of the redistribution of nitrogen from consumed amino acids to the *de novo* synthesis of N‐containing compounds. Conversely, in nitrogen‐poor medium, the direct incorporation of exogenous leucine into biomass was severely limited (30%). It has recently been demonstrated that during growth in a complex nitrogen resource, yeasts catabolized a large portion of the consumed amino acids to provide an intracellular nitrogen pool that was further used in combination with CCM precursors for the *de novo* synthesis of proteinogenic amino acids corresponding to anabolic requirements (Crépin *et al*., 2017). Our latter observations suggested that to overcome nitrogen deficiency, yeast strongly favours the catabolism of all the available amino acids to provide this intracellular pool of nitrogen for improved management of a limited nitrogen resource.

**Figure 7:**
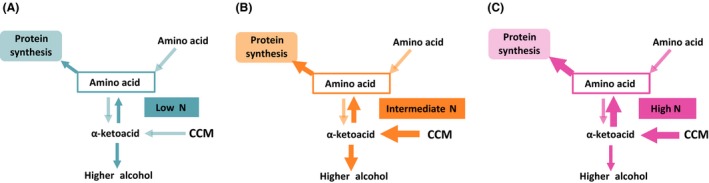
Distribution of fluxes leading to the formation of higher alcohols on the basis of the initial nitrogen content: SM70 (A), SM250 (B) and SM425 (C).

Surprisingly, the total production of higher alcohols changed differently depending on nitrogen availability, as it exhibited a maximal level at the moderate dose of nitrogen (250 mg l^−1^), in agreement with previous studies (Jiménez‐Martí *et al*., 2007; Vilanova *et al*., 2012; Mouret *et al*., 2014; Rollero *et al*., 2015). From the integrated analysis of carbon mass balances and labelling patterns, an explanatory model elucidating the effect of the nitrogen availability on the redistribution of fluxes around the α‐ketoisovalerate and the α‐ketoisocaproate nodes could be proposed. When the nitrogen resource is limited (70 mg l^−1^, Fig. 7A), a substantial part of the consumed amino acids is catabolized to provide intracellular nitrogen; the subsequent release of α‐ketoacids combined with the low anabolic requirements for leucine and valine results in a limited formation of the α‐ketoisovalerate and α‐ketoisocaproate intermediates from CCM and a moderate production of higher alcohols, which removes the surplus of α‐ketoacids precursors. When the nitrogen content increases from 70 to 250 mg l^−1^ (250 mg l^−1^, Fig. 7B), the synthesis of α‐ketoacids through the CCM increases to fulfil the greater requirements of these precursors for anabolism (three times higher). This response caused an excess of α‐ketoacids, which results in a marked increase in the flux towards the formation of higher alcohols. Finally, for a greater increase in nitrogen content, and thus a greater increase in the anabolic demand (425 mg l^−1^, Fig. 7C), the intracellular α‐ketoacids were to a large extent directed towards the synthesis of amino acids at the expense of higher alcohol formation, which results in a decreased flux in the formation of aromas compared with the previous conditions. Further analyses of changes in the intracellular α‐ketoacids content according to assimilable nitrogen availability should help to support and strengthen our hypothesis. Nevertheless, in any case, this view is in agreement with the nitrogen‐dependent regulation of the biosynthetic pathways of amino acids mediated by the derepressor Gcn4: the accumulation of uncharged tRNAs, which especially occurs in nitrogen‐starved cells, results in the activation of *GCN2* that mediates the expression of *GCN4* (Hinnebusch, 1997; Tate *et al*., 2017). In our context, the increase in protein formation to sustain yeast growth with nitrogen availability could modify the balance between synthesis and use of tRNAs, and thus activate the *GCN4*‐mediated derepression of genes responsible for the biosynthesis of amino acids, providing an excess of α‐ketoacid intermediates.

It is noteworthy that the isotopic enrichment profiles were similar between higher alcohols and their corresponding branched acids. This is consistent with the existence of shared precursors for the synthesis of these compounds. However, nitrogen availability affected the total production of acids and higher alcohols differently. The redox state of cells may explain this differential response, as the formation of both these compounds involves many dehydrogenases (Schoondermark‐Stolk *et al*., 2005). The activity of these enzymes depends on redox cofactor availability, which in turn depends on the amount of biomass produced, which requires the consumption of NADPH and NAD^+^. In nitrogen‐poor medium, the growth is limited, resulting in an excess of NAD^+^ that can be regenerated through the synthesis of acids via the Ehrlich pathway (Hazelwood *et al*., 2008). In agreement with this, Bloem *et al*. (2015) have shown that a perturbation in the availability of redox cofactors resulted in a decrease in the production of these volatiles compounds.

The impact of phytosterols on the biosynthetic pathways of higher alcohols and acids was more complex: more alcohols were produced overall at high phytosterol content, but no real impact on acids was detected. Our results also highlighted the interaction between nitrogen and lipids having a more pronounced effect on isobutanol than on isoamyl alcohol. Moreover, the quantitative analysis of flux distributions in the metabolic network revealed that α‐ketoisovalerate was largely directed towards the synthesis of α‐ketoisocaproate, with its conversion into isobutanol and isobutyric acid accounting for less than one‐third of the total flux. As a consequence, the different responses of the formation of isobutyl derivatives and isoamyl compounds to nitrogen and lipid availability may be explained by the regulating effect of the synthesis of α‐ketoisocaproate from α‐ketoisovalerate. This synthesis involves *LEU4*,* LEU2* and *LEU1*, which are controlled by the complex Leu3p‐α‐isopropylmalate, which in turn is sensitive to the level of nitrogen (Kohlhaw, 2003). Thus, it can first act as an inhibitor in leucine‐rich medium or as an activator when α‐isopropylmalate (an intermediate in the biosynthesis of leucine) accumulates in medium deficient in leucine (Sze *et al*., 1992). In addition, the expression of *LEU3* is regulated by the general amino acid control system mediated by the transcription factor Gcn4 (Zhou *et al*., 1987), which also controls the expression of genes involved in the biosynthetic pathway of leucine (*ILV3*,* LEU4*,* BAT1* and *BAT2*) (Hsu *et al*., 1982; Hu and Kohlhaw, 1995). Finally, differences in the responses of isoamyl alcohol and isobutanol production have previously been reported in the case of redox perturbation, where the availability of acetyl‐CoA was modified according to the redox status of the cell (Celton *et al*., 2012). These differences were explained by the modulation of the conversion of α‐ketoisovalerate and α‐ketoisocaproate by the intracellular content of acetyl‐CoA, which is required for the first step of this metabolic pathway (Roeder and Kohlhaw, 1980). Thus, a change in the availability of acetyl‐CoA triggered by modifications in the phytosterol content could explain why the response to change was more significant for the formation of isobutyl compound than for the production of isoamyl derivatives.

Overall, this study highlights the potential of isotopic filiation approaches to provide new insight into the metabolism of nitrogen‐containing and aroma compounds. A future challenge will be to predict and to adjust the formation of aroma compounds, which remains a complex task due to the fact that their production depends not only on the amount and nature of nutrients but also on the yeast strain used to perform the fermentation. The combination of the knowledge gained at the intracellular level of kinetics data, such as specific rates and dynamics of production, will pave the way for the development of an explanatory, dynamic and predictive model of the production of fermentative aromas.

## Experimental procedure

### Yeast strains, fermentation conditions and sampling

All experiments in this study were performed with the commercial wine strain *Saccharomyces cerevisiae* Lalvin EC1118^®^ (Lallemand SA, Montreal, Canada). Fermentation flasks were inoculated with 10 g hl^−1^ active dry yeast that had been previously rehydrated for 30 min at 37°C in a 50 g l^−1^ glucose solution (1 g of dry yeast diluted in 10 ml of this solution).

Fermentations were carried out in 330 ml reactors equipped with fermentation locks to maintain anaerobiosis, obtained by bubbling argon into the medium at 24°C with continuous magnetic stirring (150 rpm). Cultures were performed in a synthetic medium (SM) that simulates standard grape juice (Bely *et al*., 1990) and is characterized by a low pH (3.3) and high sugar content (100 g l^−1^ glucose and 100 g l^−1^ fructose). The nitrogen source was composed of ammonium chloride and amino acids (Table SD3). SM was initially supplemented with two different concentrations of phytosterols (85451, Sigma‐Aldrich, Saint‐Louis, MO, USA): 2 and 8 mg l^−1^, to satisfy the lipid requirements of yeast cells during anaerobic growth. The stock solution was composed of 15 g l^−1^ phytosterols in a mixture of Tween 80 and ethanol (1:1, v/v). The labelled nitrogen sources used were obtained from Euriso‐top^®^ (Cambridge Isotope Laboratories, Inc., Tewksbury, MA, USA): L‐valine (U‐^13^C5, 97–98%, CLM‐2249) and L‐leucine (U‐^13^C6, 97–99%, CLM‐2261).

Each condition was tested in duplicate. By measuring the change in weight throughout the process, the amount of CO_2_ released was determined and the fermentation progress was monitored (Rollero *et al*., 2015). Samples (2 aliquots of 6 ml) were taken throughout the fermentation process at stages corresponding to partial (^1^/_2_ and ¾) or complete consumption of the nitrogen resource and at the end of fermentation, which were referred to as N_1/2_, N_3/4_, N_T_ and EF respectively. Cells were pelleted by centrifugation (2000 × *g*, 5 min and 4°C) and washed twice with distilled water. Supernatants and cell pellets were stored at −20°C until analysis.

### Quantification of consumed and proteinogenic amino acids

Dry weights were determined by weight difference. Culture (10 ml) was filtered through preweighed nitrocellulose filters (pore size 0.45 μm, Millipore, Molsheim, France). The filters were twice washed with 20 ml of distilled water and dried at 105°C for 48 h before weighing (i.e. until no further change in weight was observed).

Residual ammonium ions in the supernatant were assayed spectrophotometrically using an Enzytec™ kit (5380, Enzytec™ Grosseron SAS, Coueron, France) according to the manufacturer's instructions. Before quantifying the residual amino acids, molecules with high molecular weights were removed from the supernatants by the addition of one volume of 25% (w/v) sulfosalicylic acid solution to four volumes of sample, followed by incubation at 4°C for 1 h. After centrifugation (4°C, 10 min, 3000 × *g*), the sample was filtered through a 0.22‐μm pore‐size Millipore nitrocellulose membrane. The amino acid concentrations were determined using a specific amino acid analyser (Biochrom 30, Biochrom, Holliston, MA, USA) combining ion‐exchange chromatography and spectrophotometric detection after reaction with ninhydrin, as previously described (Crepin *et al*., 2012).

We used the protein fraction of the biomass and the relative concentrations of amino acids within proteins previously determined by Crépin *et al*. (2017). The percentage of each amino acid in the proteins was further calculated from these data by dividing the measured amount of each amino acid (mg l^−1^) by the total amount of amino acids measured in the protein extract (sum in mg l^−1^). To assess the concentration of each proteinogenic amino acid in the culture (mg l^−1^), these percentages were then multiplied by the concentration of total protein in the culture (mg l^−1^), which was the product of the protein content of the biomass and the dry weight.

### Measurement of the isotopic enrichment of intracellular amino acids

#### Biomass hydrolysis

The determination of the isotopic enrichment of proteinogenic amino acids was performed using 1–2 mg dried biomass prehydrolysed in 1200 μl of 6 M HCl for 16 h at 105°C. The sample, after the addition of 800 μl of distilled water, was centrifuged at 3000 × *g* for 5 min to remove cellular debris. The supernatant was distributed into four 400 μl fractions, which were further dried at 105°C until they reached a syrup‐like state (4–5 h). These fractions were then utilized for amino acid derivatization using two different agents.

The *ethylchloroformate (ECF) derivatization* procedure was modified from Christensen and Nielsen (1999). The dried hydrolysate was dissolved in 200 μl of 20 mM HCl and 133 μl of a pyridine–ethanol mixture (1:4). The amino acids in this mixture were derivatized by adding 50 μl of ECF. The derivatives were extracted into 500 μl of dichloromethane. For analysis, the upper organic phase was collected after 4 min of centrifugation at 9000 × *g* and injected directly into a GC/MS system.

The *N,N‐dimethylformamide dimethyl acetal (DMF/DMA)* derivatization procedure was modified from Christensen and Nielsen (1999). The dried hydrolysate was dissolved in 50 μl of methanol and 200 μl of acetonitrile, and the mixture was derivatized by adding 300 μl of DMF/DMA. The extract was directly injected into the GC/MS.

#### GC‐MS analysis

Samples were then analysed with a Hewlett Packard 6890 gas chromatograph (Agilent Technologies, Santa Clara, CA, USA) equipped with a CTC Combi PAL Autosampler AOC‐5000 (Shimadzu, Columbia, SC, USA) and coupled to an HP 5973 mass spectrometer. The instrument was controlled and the data were analysed using HP G1701DA ChemStation software. The gas chromatograph was fitted with a 30 m × 0.25 mm DB‐17 ms column with a film thickness of 0.15 μm (Agilent Technologies). Compounds obtained from the ECF and DMF/DMA derivatizations were separated using the analytical conditions described by Crépin *et al*. (2017).

The MS was operated in selected ion monitoring (SIM) mode with positive ion electron impact at 70 eV using the characteristic ions of amino acid fragments reported in Crepin *et al*., (2017). For each amino acid fragment, the outcome of the analysis was a cluster of intensities corresponding to its different mass isotopomers. These data were subsequently processed using the IsoCor software recently developed by Millard *et al*. (2012) to correct for natural labelling and to assess the isotopic enrichment of each amino acid, which is defined as the fraction of labelled compound with respect to its total amount in proteins (expressed in percentage).

### Quantification and isotopic enrichment of volatile compounds

The labelled volatile compounds were extracted according to the method described by Rollero *et al*. (2015), using dichloromethane after the addition of deuterated internal standards. The extracted volatile molecules were separated using a Hewlett Packard 6890 gas chromatograph (Agilent Technologies) equipped with a 30 m × 0.25 mm Phenomenex ZB‐WAX‐fused silica capillary column with a 0.25 μm film thickness (Agilent Technologies) and helium as the carrier gas using the procedure previously described by Rollero *et al*. (2015). Compounds were detected using an HP 5973 mass spectrometer in SIM mode with positive ion electron impact at 70 eV. For the quantification and the determination of the labelling patterns of volatile compounds we used the ion clusters reported in Crepin *et al*. (2017). These ion clusters were selected on the basis of their high signal‐to‐noise ratio and low interference from other compounds. The concentration of each volatile molecule was quantified from the sum of the intensities of the corresponding ion cluster. In parallel, for each ion clusters, the intensities were corrected for natural labelling using isocor software (Millard *et al*., 2012) and processed to assess to the isotopic enrichment of volatile compounds, which is defined as the fraction of labelled molecule with respect to its total production (expressed in percentage).

### Outline of calculations for flux quantification

The labelled fraction of a proteinogenic amino acid or a volatile compound was calculated by multiplying its concentration in mM by its isotopic enrichment. The difference from the total amount of the compound corresponded to the unlabelled part. Fluxes in the metabolic reactions involved in the synthesis of a target compound (proteinogenic amino acid or volatile molecule) from a labelled nitrogen source were quantified by dividing the labelled fraction of the compound by the total amount of consumed labelled molecule in mM (Tables SD1 and SD2).

## Conflict of Interest

None declared.

## Supporting information


**Table S1.** Summary of the data set obtained during fermentation with ^13^C valine.
**Table S2.** Summary of the data set obtained during fermentation with ^13^C leucine.
**Table S3.** Percentage of yeast assimilable nitrogen provided by each nitrogen sources.
