# Supplementary material for: Quantitative 13C‐isotope labelling‐based analysis to elucidate the influence of environmental parameters on the production of fermentative aromas during wine fermentation
Source: Microb Biotechnol. 2017 Jul 11;10(6):1649–62. doi: 10.1111/1751-7915.12749 (PMC5658611; doi:10.1111/1751-7915.12749)
Supplement: Supplementary file 1 — Table S1. Summary of the data set obtained during fermentation with 13C valine. Table S2. Summary of the data set obtained during fermentation with 13C leucine. Table S3. Percentage of yeast assimilable nitrogen provided by each nitrogen sources. [file MBT2-10-1649-s001.docx]

Table S1 : Summary of the data set obtained during fermentation with ^13^C valine

Table S2 : Summary of the data set obtained during fermentation with ^13^C leucine

Table S3: Percentage of yeast assimilable nitrogen provided by each nitrogen sources

|  | % YAN for each N sources |
| --- | --- |
| Tyrosine | 0.3% |
| Tryptophane | 3.0% |
| Isoleucine | 0.8% |
| Aspartate | 1.1% |
| Glutamate | 2.8% |
| Arginine | 22.0% |
| Leucine | 1.3% |
| Threonine | 2.2% |
| Glycine | 0.8% |
| Glutamine | 23.6% |
| Alanine | 5.6% |
| Valine | 1.3% |
| Methionine | 0.7% |
| Phenylalanine | 0.8% |
| Serine | 2.5% |
| Histidine | 0.7% |
| Lysine | 0.8% |
| Cysteine | 0.4% |
| NH4Cl | 29.3% |
